# SERS Mixture Recognition from Pure-Substance Spectra via Component Evidence Learning and Two-Stage Inference

**DOI:** 10.3390/molecules31091412

**Published:** 2026-04-24

**Authors:** Li Fan, Daoyu Lin, Liang Shen, Junjun Guo, Ting Lian, Yazhou Qin

**Affiliations:** 1 Aerospace Information Research Institute, Chinese Academy of Sciences, Beijing 100094, China; 2 Key Laboratory of Target Cognition and Application Technology (TCAT), Aerospace Information Research Institute, Chinese Academy of Sciences, Beijing 100190, China; 3 School of Electronic, Electrical and Communication Engineering, University of Chinese Academy of Sciences, Beijing 100049, China; 4 Keqiao Branch, Shaoxing Municipal Public Security Bureau, Shaoxing 312000, China; 5 Key Laboratory of Drug Prevention and Control Technology of Zhejiang Province, Zhejiang Police College, 555 Binwen Road, Binjiang District, Hangzhou 310053, China

**Keywords:** surface-enhanced Raman spectroscopy (SERS), spectral mixture recognition, component evidence learning, two-stage inference

## Abstract

Surface-enhanced Raman spectroscopy (SERS) is a powerful tool for molecular analysis, yet the recognition of mixed spectra remains challenging because severe peak overlap makes mixture-specific data expensive to acquire and difficult to cover exhaustively. Current machine-learning approaches often rely on labeled mixture datasets, synthetic mixed spectra, or prior component-matching schemes, making their performance strongly dependent on task-specific mixture data. A pure-spectrum-trained framework for SERS mixture recognition is presented here based on component evidence learning and two-stage inference. Using paraquat, thiram, and tricyclazole as representative target compounds, the framework learns reusable constituent-level evidence directly from pure-substance spectra and converts it into mixture-category predictions within a unified recognition model. This design avoids mixture-specific parameter training while enabling direct recognition of binary and ternary mixtures. Experiments on SERS spectral datasets yielded a mixture recognition accuracy of 98.58%. The results show that pure-substance spectral learning can support accurate recognition of complex SERS mixtures and provide a scalable strategy for mixture analysis when labeled mixture data are limited.

## 1. Introduction

Surface-enhanced Raman spectroscopy (SERS) has become an important analytical tool for chemical and biochemical sensing because it combines high sensitivity with molecular fingerprint specificity, enabling rapid and label-free characterization of complex samples. In recent years, SERS applications have expanded well beyond conventional single-analyte analysis and increasingly targeted chemically heterogeneous systems, including multiplex detection of bacterial lipopolysaccharides, differential diagnosis based on multiple sepsis-related biomarkers, physiological-state classification from sweat metabolites, and simultaneous analysis of multiple metabolites in mixed solutions [1,2,3,4]. These developments indicate that practical SERS analysis is moving from idealized pure-compound scenarios toward more realistic multicomponent environments.

As SERS is applied to increasingly complex samples, mixture analysis has emerged as a major analytical challenge. In many realistic sensing scenarios, the measured spectrum is generated not by a single isolated species, but by several coexisting compounds interacting competitively with the enhancing substrate. Under such conditions, competitive adsorption, spectral overlap, peak attenuation, concentration imbalance, and matrix interference may distort or obscure the spectral fingerprints of individual constituents. Recent studies on trace-target sensing in complex SERS mixtures have shown that these effects substantially reduce the reliability of qualitative and quantitative interpretation, particularly when weak target signals are embedded in strong interfering backgrounds [5,6,7]. As a result, recognizing mixed spectra is substantially more difficult than recognizing pure spectra and remains a central obstacle for practical SERS-based sensing.

A conventional strategy for handling such complexity is to formulate the problem as spectral decomposition or unmixing. In Raman spectroscopy, this class of methods seeks to decompose an observed mixed spectrum into latent spectral signatures and their corresponding abundances, usually under linear or weakly nonlinear mixing assumptions. These approaches have long been regarded as promising for molecular visualization and mixture analysis, but their performance depends strongly on assumptions regarding endmembers, mixing behavior, and signal quality [8]. More recently, Georgiev et al. reformulated Raman unmixing as a self-supervised learning problem and showed that physics-constrained autoencoders can infer abundance-like latent representations and reconstruct complex mixed spectra more robustly than conventional unmixing methods [9]. Related work such as SSNet further demonstrated that self-supervised unmixing can improve qualitative sensitivity in complex SERS matrices by separating target signals from background interference [5]. Although these studies confirm the value of decomposition-oriented learning for complex-spectrum analysis, their primary objective remains signal separation or abundance-related representation learning rather than direct recognition of discrete mixture categories.

In parallel with decomposition-based approaches, Raman and SERS analysis has increasingly shifted toward data-driven representation learning. Recent methodological studies and reviews have shown that machine learning and deep neural networks can reduce dependence on handcrafted preprocessing and manually designed spectral descriptors by learning hierarchical representations directly from raw or minimally processed spectra [10,11,12,13]. Within the specific context of mixture analysis, several studies have demonstrated that deep learning can achieve strong predictive performance when representative mixture data are available. For example, DeepCID showed that convolutional neural networks can learn discriminative features for component identification in Raman spectra of mixtures and outperform conventional machine-learning baselines [14]. DeepRaman further improved component identification by combining a pseudo-Siamese architecture with spatial pyramid pooling [15]. Other supervised studies on portable Raman analysis and complex organic mixtures have likewise shown that deep models can identify compounds, classify mixtures, or estimate constituent proportions when the relevant compositional space is sufficiently represented during training [16,17]. These studies collectively establish deep learning as a practical route for mixture analysis once the target mixture distribution is adequately covered [18].

However, the dominant supervised paradigm remains strongly dependent on mixture-specific data. As the number of candidate components increases, the number of possible binary, ternary, and higher-order combinations grows combinatorially. Collecting sufficiently representative labeled spectra for all relevant combinations therefore becomes increasingly costly and, in many realistic sensing scenarios, infeasible. This limitation is evident in recent SERS studies on microplastic mixtures, mixed-antibiotic recognition, and quantitative analysis of mixed analytes, where model performance remains closely tied to the availability of task-specific mixture datasets [6,7,19]. In other words, although supervised models can perform well within a predefined compositional space, their performance still depends strongly on whether representative mixture categories are covered during model development.

To reduce dependence on experimentally collected mixture datasets, recent studies have increasingly explored the use of single-component information. SpecRecFormer represents a notable example of this direction. Rather than directly predicting mixture categories, it compares mixed spectra with known single-component reference spectra using a dual-channel convolutional neural network and a Transformer module, and then identifies candidate components through adaptive thresholding [20]. This framework demonstrates that single-component spectra can provide useful prior knowledge for mixture analysis. However, it still depends on synthetic mixture augmentation during training, and its decision process remains based on pairwise similarity evaluation followed by threshold-based component selection [21]. As a result, the method is fundamentally oriented toward component matching rather than direct mixture-category recognition.

A conceptually related but more advanced direction is pure-spectrum-driven transfer learning. Tan et al. showed that models trained solely on pure compound SERS spectra can be transferred to binary, ternary, and quaternary mixtures for sequential identification and quantification [22]. This result is highly relevant because it demonstrates that pure-spectrum training can capture reusable chemical information for downstream mixture analysis without requiring mixture-specific training sets. However, the framework remains sequential and multi-stage: one set of models is first used to identify likely components, after which additional models are selected to estimate ratios and concentrations. Although highly effective for component-wise analytical workflows, such a design still separates component identification from downstream compositional analysis, rather than directly mapping pure-spectrum learning to mixture-category recognition within a unified model.

Taken together, the current literature reveals a clear methodological gap. Unmixing-based methods provide interpretable decomposition of complex spectra, but they are not primarily designed for direct mixture-category recognition. Supervised deep learning models can achieve high predictive accuracy, but they depend on labeled mixture datasets and scale poorly as the combinatorial mixture space expands. Single-component-driven and transfer-learning-based methods show that reusable knowledge can be extracted from pure spectra, yet current formulations remain largely decomposition-oriented, component-matching-oriented, sequential, or dependent on synthetic mixtures. Therefore, direct mixture-category recognition based on reusable component-level evidence learned from pure SERS spectra remains insufficiently explored, particularly under settings that avoid sequential component-wise analysis, synthetic mixture augmentation, and mixture-specific parameter training [23].

In this work, a pure-spectrum-trained framework for SERS mixture recognition is established based on component evidence learning and two-stage inference. Unlike existing methods that rely on spectral decomposition, sequential identification followed by quantification, or component-matching strategies supported by synthetic mixtures, the present method is designed for direct mixture-category recognition within a unified model. The model learns discriminative spectral representations together with reusable component-level evidence from pure spectra and converts these evidence scores into final mixture decisions through a two-stage inference strategy. Binary mixture candidates are first evaluated by set-likelihood inference, after which a residual-based ternary refinement step determines whether the input spectrum is better assigned to a ternary mixture category. In this way, reusable single-component knowledge is directly connected to the recognition of binary and ternary mixtures without requiring mixture samples for parameter training.

## 2. Results and Discussion

### 2.1. Characterization of SERS

**Figure 1:**
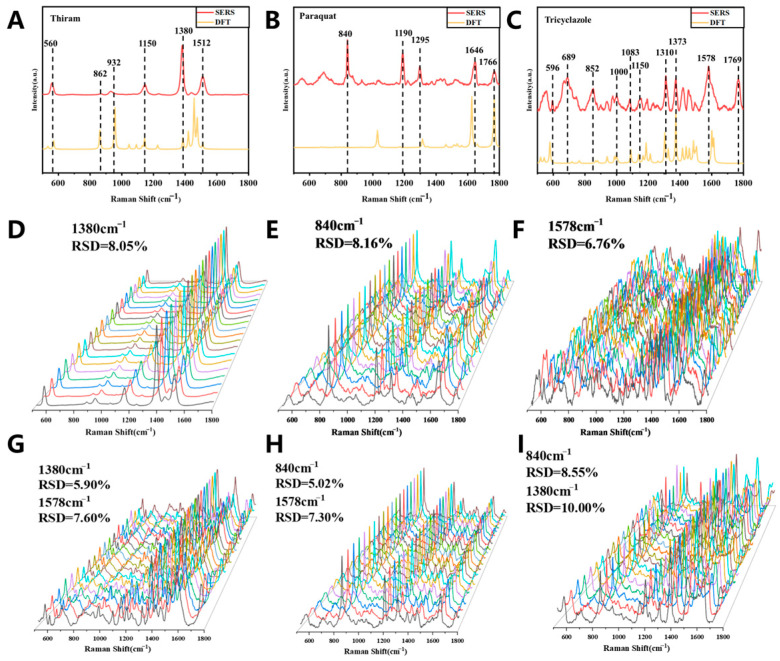
SERS and DFT calculated Raman spectra of (**A**,**D**) paraquat, (**B**,**E**) thiram, and (**C**,**F**) tricyclazole, (**G**) thiram and tricyclazole, (**H**) paraquat and tricyclazole, (**I**) paraquat and thiram.

We first analyzed the SERS spectra of three pure substances. The SERS and DFT-calculated Raman spectra of paraquat, thiram, and tricyclazole are shown in Figure 1 and Tables S1–S3. The theoretical calculations were performed using the density functional theory (DFT) B3LYP method with the 6-311++G(d,p) basis set. In practical testing, different molecular orientations can cause certain characteristic vibrational modes to become prominent, resulting in discrepancies in the relative intensities between the experimental spectra and calculated spectra for some vibrational modes. To correct the errors introduced by the harmonic approximation, the entire Raman shift range was multiplied by corresponding correction factors (Tables S1–S3) to align with the experimentally observed SERS peak positions, thereby completing the calibration of the DFT-calculated spectra.

As we can see, the main characteristic peaks of paraquat were observed at 840, 1029, 1190, 1298, 1646, and 1766 cm^−1^. Specifically, the band at 840 cm^−1^ was assigned to the pyridine ring breathing vibration and the C–N stretching vibration between the pyridine ring and the methyl group. The peak at 1029 cm^−1^ was attributed to the C=C rocking vibration of the pyridine ring. The band at 1190 cm^−1^ corresponded to the C–H rocking vibration of the pyridine ring, while the peak at 1298 cm^−1^ was assigned to the in-plane C–H rocking vibration of the pyridine ring. The band at 1646 cm^−1^ was related to the C–H rocking vibration of the pyridine ring and the C=C stretching vibration between the pyridine rings. The peak at 1766 cm^−1^ was attributed to the C=C stretching vibration of the pyridine ring as well as the C=C stretching vibration between the two pyridine rings.

The characteristic peaks of thiram were mainly located at 560, 932, 1380, and 1512 cm^−1^. Among them, the band at 560 cm^−1^ was assigned to C=S rocking, C–S rocking, and C=N rocking vibrations. The peak at 932 cm^−1^ corresponded to C=S stretching and C–N stretching vibrations. The band at 1380 cm^−1^ was attributed to C=N stretching and C–H rocking vibrations of the methyl group, while the peak at 1512 cm^−1^ was assigned to the C–H rocking vibration of the methyl group.

For tricyclazole, the main characteristic peaks appeared at 596, 988, 1000, 1090, 1193, 1303, 1403, 1420, and 1589 cm^−1^. The band at 596 cm^−1^ was assigned to the benzene ring breathing vibration. The peak at 988 cm^−1^ corresponded to triazole ring breathing and in-plane bending of the benzene ring. The band at 1000 cm^−1^ was attributed to N–N stretching of the triazole ring and C–H rocking of the methyl group attached to the benzene ring. The peak at 1090 cm^−1^ was assigned to benzene ring breathing and C–H rocking of the methyl group attached to the benzene ring. The band at 1193 cm^−1^ was related to C–H rocking of the benzene ring and C–C stretching of the methyl group attached to the benzene ring. The peak at 1303 cm^−1^ was assigned to C–H rocking of the benzene ring and C=N stretching of the triazole ring. The band at 1403 cm^−1^ corresponded to C–H rocking of the benzene ring and C–N stretching between the benzene ring and the triazole ring. The peak at 1420 cm^−1^ was attributed to C–H rocking of the methyl group attached to the benzene ring. The band at 1589 cm^−1^ was assigned to C=C stretching of the benzene ring and in-plane C–H rocking of the benzene ring.

Figure 1D,F show the SERS detection results of three substances at 20 randomly selected sites. For thiram, the RSD of its characteristic peak at 1380 cm^−1^ is 8.05%. For paraquat, the RSD of its characteristic peak at 840 cm^−1^ is 8.16%. For tricyclazole, the RSD of its characteristic peak at 1578 cm^−1^ is 6.76%. Figure 1G–I represent the results obtained from pairwise mixing followed by SERS testing. It can be observed that the RSD values are all within 10%, demonstrating the good stability of the method. The SERS test results of the three mixed substances are shown in Figure S8, demonstrating excellent stability as well.

**Figure 2:**
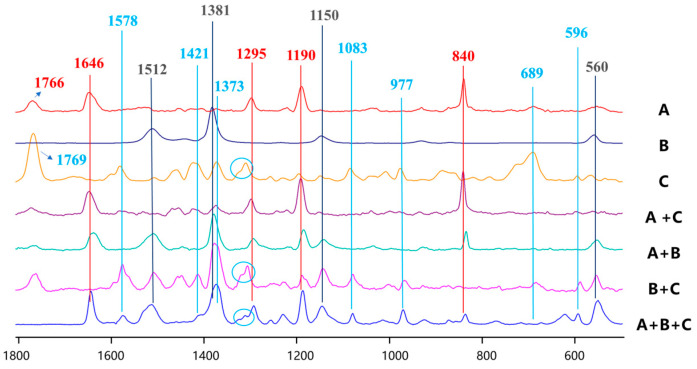
SERS spectra of (A) paraquat, (B) thiram, (C) tricyclazole, (A+C) paraquat + tricyclazole, (A+B) paraquat + thiram, (B+C) tricyclazole + thiram, and (A+B+C) paraquat + tricyclazole + thiram.

Figure 2 shows the SERS detection results of the three substances and their mixtures under different combination conditions. The red color indicates the characteristic peak positions of paraquat, the black color represents the characteristic peak positions of thiram, and the blue color denotes the characteristic peak positions of tricyclazole. It can be observed from the figures that when the three substances are mixed, their SERS spectra do not exhibit simple linear superposition, and some characteristic peaks may be masked by other components in the mixture. When paraquat is mixed with tricyclazole (A+C in Figure 2), due to the particularly strong SERS signal of paraquat, the characteristic peaks of paraquat (840 cm^−1^, 1190 cm^−1^, 1295 cm^−1^, and 1646 cm^−1^) are predominantly exhibited. Meanwhile, the characteristic peak of tricyclazole at 689 cm^−1^ becomes submerged, with only a relatively weak characteristic peak appearing near 1421 cm^−1^. Consequently, the SERS detection of the mixture imposes higher requirements on the classification and recognition performance of the model.

### 2.2. Training Stability and Pure-Spectrum Representation Analysis

#### 2.2.1. Loss Curves

**Figure 3:**
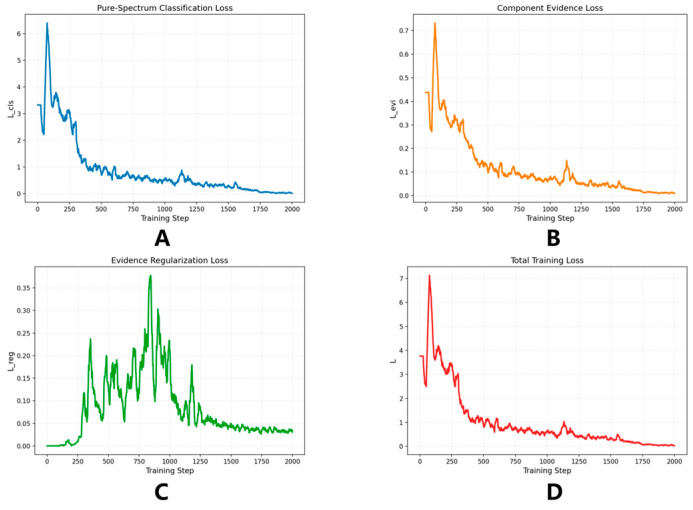
Training curves of the pure-spectrum classification loss (**A**), component evidence loss (**B**), evidence regularization loss (**C**), and total loss (**D**). The results show stable optimization and overall convergence of the proposed dual-branch framework under pure-spectrum supervision.

To evaluate the optimization behavior of the proposed dual-branch framework, the training curves of the pure-spectrum classification loss, component evidence loss, evidence regularization loss, and total loss are shown in Figure 3. Overall, all loss terms exhibit stable training behavior, indicating that the framework can be effectively optimized under pure-spectrum supervision and that the shared encoder can support both pure-spectrum discrimination and component-level evidence learning without evident optimization conflict.

As shown in Figure 3A, the pure-spectrum classification loss decreases rapidly at the beginning of training and then gradually stabilizes at a low level, suggesting that the classification branch quickly establishes separable representations for the three pure components. A similar downward trend is observed for the component evidence loss in Figure 3B, indicating that the evidence branch progressively improves its ability to assign high evidence to the target component while suppressing non-target components. This behavior is important because downstream mixture recognition depends on the transferability of component-level evidence rather than on direct supervision from mixture labels.

By contrast, the evidence regularization loss in Figure 3C first increases and then gradually decreases. This pattern is consistent with the role of the regularizer: as the confidence of the evidence branch increases during early training, the magnitude of the evidence logits grows and activates the clipping-based constraint; with continued optimization, the regularizer gradually limits excessively large evidence responses, leading to a subsequent decline. As shown in Figure 3D, the total loss exhibits a clear convergence trend, with a pronounced decrease in the early stage and a smoother decline thereafter. Importantly, the temporary increase in the regularization term does not disrupt overall optimization.

In summary, Figure 3 shows that the proposed framework can be trained in a coordinated and stable manner using only pure spectra. The classification branch learns discriminative spectral representations, the evidence branch learns transferable component-level probabilities, and the regularization term helps maintain calibrated evidence responses, thereby providing the optimization basis for subsequent binary and ternary mixture recognition.

#### 2.2.2. t-SNE Visualization of Pure-Spectrum Embeddings

t-distributed stochastic neighbor embedding (t-SNE) [24] was used to visualize the latent features extracted from pure SERS spectra. Because the framework is trained on pure spectra and then applied to mixture recognition, the organization of the pure-spectrum feature space is directly relevant to downstream inference.

**Figure 4:**
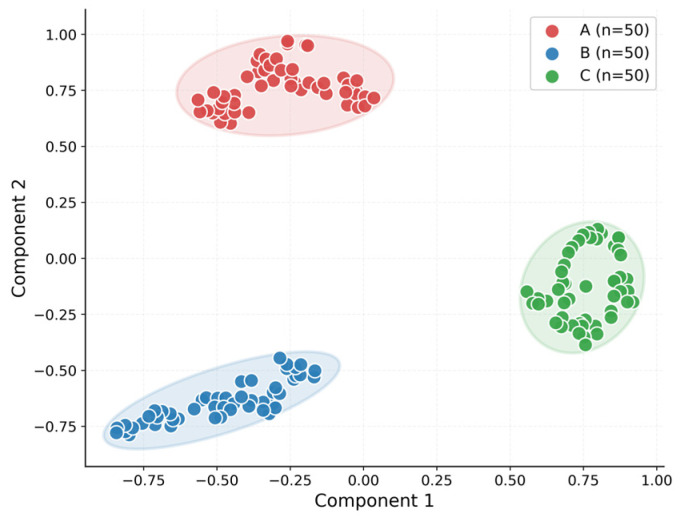
t-SNE visualization of latent embeddings for representative pure SERS spectra of paraquat (A), thiram (B), and tricyclazole (C). Fifty spectra from each pure-substance test class were projected into a two-dimensional space using features extracted by the trained shared encoder. The three classes form clearly separated clusters with limited overlap.

In this study, latent features were extracted from the shared encoder of the best-performing model and projected into a two-dimensional space using t-SNE. Paraquat, thiram, and tricyclazole were denoted as A, B, and C, respectively, and 50 spectra were randomly selected from each pure-spectrum test class for visualization. The resulting t-SNE map is shown in Figure 4.

As shown in Figure 4, the embeddings of the three pure components form three clearly separated clusters with only limited overlap, indicating that the shared encoder learns discriminative latent features that preserve substance-specific differences. At the same time, the samples within each class remain relatively compact, although a certain degree of intra-class spread is still observed. This behavior suggests that the learned latent space retains stable component-specific information while remaining tolerant to moderate spectral variability.

Among the three classes, the tricyclazole cluster appears especially well separated, whereas paraquat and thiram are relatively closer but still clearly distinguishable without evident mixing. Overall, Figure 4 shows that the proposed framework organizes pure SERS spectra into a structured latent space with clear inter-class separation and satisfactory intra-class consistency, thereby supporting subsequent component evidence estimation and mixture-category inference.

#### 2.2.3. Grad-CAM of Pure Spectra

Grad-CAM [25] was used to visualize the spectral regions that contributed most strongly to pure-spectrum classification. Because downstream component evidence estimation and mixture inference both depend on the quality of the pure-spectrum representation learned by the shared encoder, this analysis provides an interpretable basis for evaluating whether the learned representations are supported by chemically meaningful characteristic bands. The resulting attribution patterns were interpreted together with the peak assignments described in Section 3.1.

**Figure 5:**
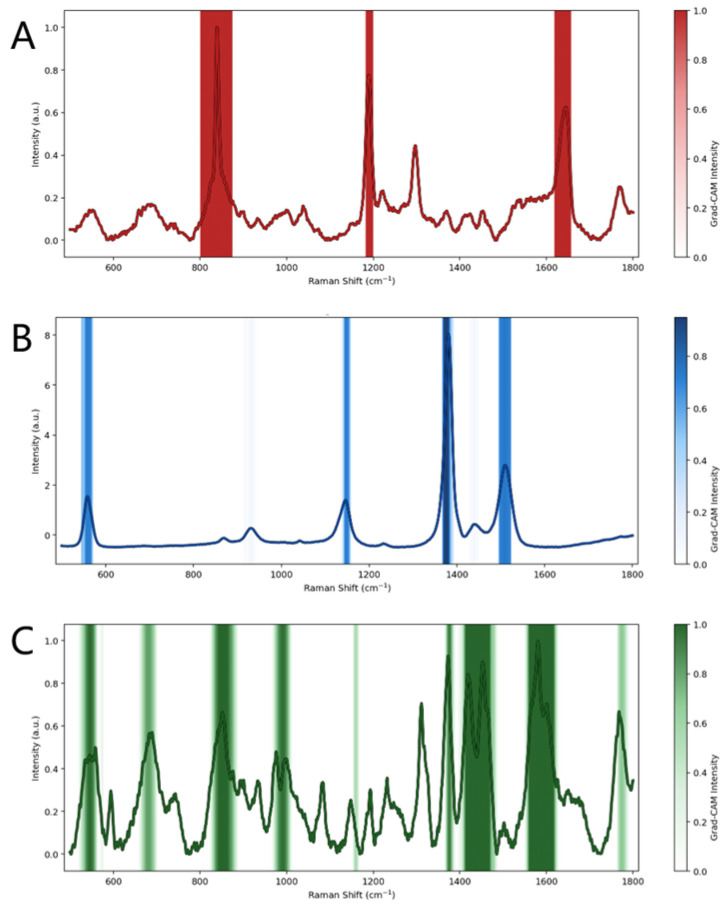
Grad-CAM visualization of representative pure SERS spectra for paraquat (**A**), thiram (**B**), and tricyclazole (**C**). The highlighted regions indicate the spectral intervals that contribute most strongly to the predicted pure-substance class. Distinct substance-dependent attribution patterns are observed across the three compounds.

Grad-CAM maps were generated for representative pure spectra of paraquat, thiram, and tricyclazole using the best-performing model. The gradients of the predicted pure-substance class score were back-propagated to the final convolutional layer of the shared encoder, and the resulting one-dimensional activation maps were normalized and overlaid on the input spectra. The results are shown in Figure 5.

As shown in Figure 5, the model exhibits distinct and chemically interpretable attribution patterns for the three substances. For paraquat (Figure 5A), the major activation regions are concentrated around the characteristic bands near 840, 1190, and 1646 cm^−1^, indicating that classification is primarily driven by a limited number of dominant discriminative bands. For thiram (Figure 5B), the activation pattern is similarly compact and mainly distributed around the characteristic regions near 560, 1380, and 1512 cm^−1^, suggesting that the model captures its essential characteristic bands. By contrast, tricyclazole (Figure 5C) shows a broader attribution pattern spanning multiple characteristic regions, including the bands near 596 cm^−1^, 988–1090 cm^−1^, 1303–1420 cm^−1^, and 1589 cm^−1^, indicating that its recognition depends on the joint contribution of several spectral intervals rather than on a few dominant peaks.

Overall, Figure 5 shows that pure-spectrum classification in the proposed framework is primarily supported by chemically relevant and substance-specific spectral regions. This result indicates that the learned discriminative representations are grounded in meaningful characteristic bands and helps explain why pure-spectrum-trained representations can support subsequent component evidence estimation and mixture-category inference.

### 2.3. Classification and Mixture Recognition Performance

#### 2.3.1. Pure-Spectrum Classification Performance

**Figure 6:**
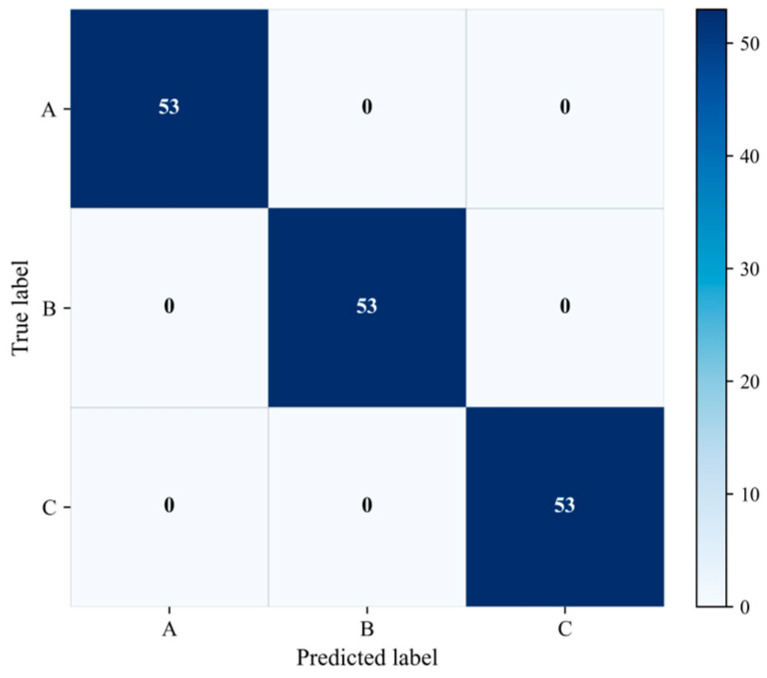
Confusion matrix for pure-spectrum classification of the three target substances. Here, A, B, and C denote paraquat, thiram, and tricyclazole, respectively. For each class, 53 spectra were randomly selected from the corresponding pure-spectrum test subset.

Pure-spectrum classification performance was evaluated using 53 samples randomly selected from each pure-spectrum test subset for paraquat, thiram, and tricyclazole, denoted as A, B, and C, respectively. The corresponding results are summarized in the confusion matrix shown in Figure 6 and the quantitative metrics listed in Table 1.

As shown in Figure 6, all test samples of the three pure substances were correctly assigned to their corresponding categories. Specifically, all 53 spectra of paraquat were classified as class A, all 53 spectra of thiram were classified as class B, and all 53 spectra of tricyclazole were classified as class C. No cross-class confusion was observed. This result indicates that the proposed framework can effectively distinguish the spectral patterns of the three target substances and that the shared encoder has learned highly discriminative pure-spectrum representations.

The quantitative metrics in Table 1 are fully consistent with the confusion matrix. The overall classification accuracy reached 1.000, and the precision, recall, and F1-score were all 1.000 at both the overall level and the class level. These results confirm that the proposed model achieved error-free classification on the selected pure-spectrum test set under the present experimental setting.

Accurate discrimination among paraquat, thiram, and tricyclazole indicates that the encoder captures stable component-specific spectral features and maintains clear inter-class separability in the latent space. This representation provides the basis for subsequent component evidence estimation and mixture-category inference.

#### 2.3.2. Mixture Recognition Performance

**Figure 7:**
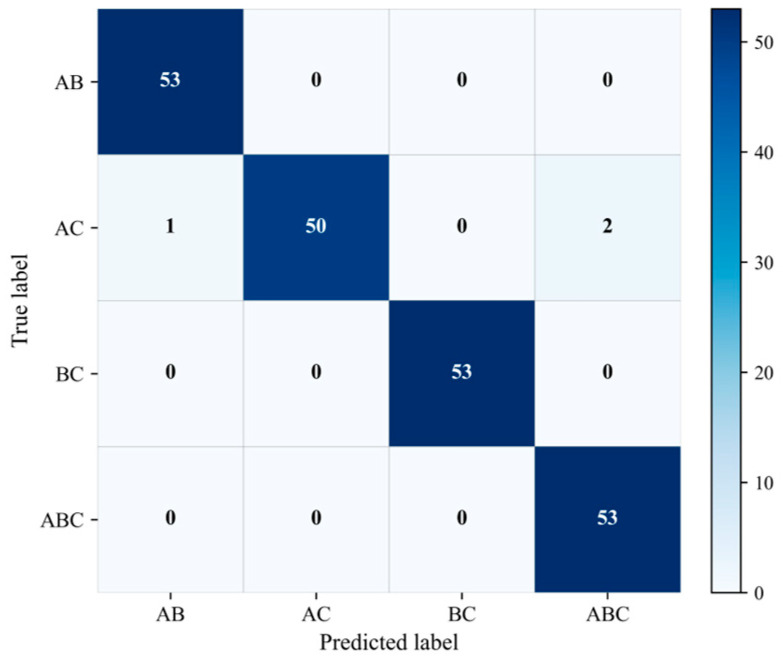
Confusion matrix for mixture recognition of binary and ternary SERS spectra. Here, A, B, and C denote paraquat, thiram, and tricyclazole, respectively. The binary mixture categories correspond to pairwise combinations of these three components, and the ternary mixture contains all three components.

Mixture recognition performance was evaluated on binary and ternary SERS spectra using a model trained only on pure-substance data. For each mixture category, 53 samples were randomly selected from the corresponding test subset. Here, A, B, and C denote paraquat, thiram, and tricyclazole, respectively, so that the binary mixtures correspond to AB, AC, and BC, whereas the ternary mixture corresponds to ABC. The results are summarized in Figure 7 and Table 2.

As shown in Figure 7, the proposed framework achieves highly accurate recognition across all four mixture categories. The AB and BC categories are both recognized without error, indicating that the corresponding binary mixtures can be reliably identified on the basis of pure-spectrum-trained representations and subsequent inference. The ABC category is also completely recognized, suggesting that the residual-based refinement stage can effectively identify spectra whose composition is more consistent with a ternary explanation than with any binary candidate.

Among the four categories, the main classification difficulty is observed for the AC mixture. According to the confusion matrix, 50 of the 53 AC samples are correctly recognized, whereas 1 sample is classified as AB and 2 samples are assigned to the ABC category. Although the number of misclassified samples is limited, this result indicates that AC is relatively more difficult to separate from the remaining candidate mixtures under the current dataset and inference setting. In particular, the assignment of a small number of AC spectra to the ternary category suggests that, for these samples, the ternary reconstruction yields a sufficiently improved fit relative to the corresponding binary hypothesis, thereby activating the residual-based refinement criterion.

The quantitative metrics in Table 2 are consistent with the confusion matrix. The proposed model achieved an overall accuracy of 0.9858, together with a macro-precision of 0.9863, a macro-recall of 0.9858, and a macro-F1 score of 0.9858. At the class level, BC achieved perfect precision, recall, and F1-score, while AB and ABC also exhibited strong recognition performance. By comparison, AC showed a slightly lower recall and F1-score owing to the small number of misclassified samples. Nevertheless, its overall recognition performance remained high, indicating that the proposed framework maintains strong discriminative ability even for the most challenging mixture category in the present dataset.

The results in Figure 7 and Table 2 show that binary and ternary SERS mixtures can be recognized accurately using representations learned only from pure-substance spectra. The high performance across all mixture categories indicates that the shared encoder learns transferable spectral features from paraquat, thiram, and tricyclazole, whereas the two-stage inference strategy converts the resulting component-level evidence into reliable mixture-category decisions.

## 3. Materials and Methods

### 3.1. Materials and Instruments

Methanol (chromatographic grade) was purchased from Merck (Darmstadt, Germany), and sodium chloride (ACS, ≥99%) was obtained from Aladdin Reagent Co., Ltd. (Shanghai, China). All reagents were used as received without further purification. Gold nanoparticles (AuNPs) were purchased from Pushi Nano Technology Co., Ltd. (Xiamen, China). Analytical standards of paraquat, thiram, and tricyclazole were obtained from COTO Standard Reagent Co (Guangzhou, China).

All aqueous solutions were prepared using ultrapure water generated by a Millipore (Burlington, MA, USA) ultrapure water system, with a resistivity of 18.2 MΩ·cm at 25 °C. A vortex mixer (Thermo Fisher Scientific, Waltham, MA, USA) was used for solution mixing. Surface-enhanced Raman scattering (SERS) measurements were carried out using a PERS-RZ1710 microscopic Raman spectrometer (PUSHI HUI NA Co., Ltd. (Xiamen, China)).

### 3.2. SERS Detection of Standards

Paraquat, thiram, and tricyclazole standards were each dissolved in methanol to prepare stock solutions at a concentration of 10^−2^ mol/L. These stock solutions were then successively diluted with methanol to obtain working solutions at 10^−3^, 10^−4^, 10^−5^, and 10^−6^ mol/L.

For mixed-sample preparation, the 10^−2^ mol/L tricyclazole and paraquat standard solutions were first mixed at a volume ratio of 1:1 and vortexed thoroughly to obtain a homogeneous binary mixture. In addition, the 10^−2^ mol/L standard solutions of tricyclazole, paraquat, and thiram were mixed pairwise at a volume ratio of 1:1 and vortexed thoroughly to obtain homogeneous binary mixtures. A ternary mixture was also prepared by mixing the 10^−3^ mol/L standard solutions of tricyclazole, paraquat, and thiram at a volume ratio of 1:1:1, followed by vortex mixing to ensure complete homogenization.

During SERS measurements, use a pipette to transfer 100 μL of the prepared standard solution or mixed standard solution into the SERS tube. Subsequently, sequentially add 100 μL of 2 mol/L NaCl solution and 100 μL of AuNPs into the glass tube containing the test solution using a pipette, and mix the solutions thoroughly using a vortex oscillator. The samples were analyzed using a PERS-RZ1710 micro-Raman spectrometer. The measurement conditions were set as follows: acquisition time, 5 s; accumulation times, 3; and laser power, 100%. A total of 1071 sets of spectral data were collected. The test data spectra are shown in Figures S1–S7.

### 3.3. Data Preprocessing

After SERS spectra were acquired, the spectral range from 500 to 1800 cm^−1^ was first selected as the fingerprint region, because this interval contains the major characteristic peaks of the investigated pesticides. To ensure consistent feature dimensionality across all samples, the spectral data were resampled to 2048 uniformly distributed feature points using linear interpolation. Savitzky–Golay smoothing (window length = 5, polynomial order = 2) was then applied to reduce random noise while preserving the sharpness and positions of the characteristic peaks. Subsequently, polynomial iterative baseline correction was employed to eliminate baseline drift. Through iterative selection of baseline points, baseline fitting, and the application of corresponding constraints, this method effectively removed baseline variations caused by scattering effects, instrumental drift, and fluorescence background, thereby enhancing the characteristic spectral signals of the samples. Finally, the spectral intensities were normalized using Min–Max normalization, followed by a power-law transformation (α = 1.5) to enhance the contrast between peaks and valleys. This preprocessing procedure reduced intensity variations arising from differences in sample concentration and measurement conditions, enabling the model to focus on spectral shape characteristics rather than absolute intensity values.

After preprocessing, the spectral data were organized into separate training, validation, and test sets for model development and evaluation. The pure-substance dataset consisted of 260 paraquat samples, 318 thiram samples, and 323 tricyclazole samples, which were divided into training, validation, and test subsets. The training set contained 157 paraquat, 215 thiram, and 220 tricyclazole samples, whereas the validation and test sets each included 50 and 53 pure-substance samples per class, respectively. To support mixture-oriented checkpoint selection and final evaluation, mixture spectra were included only in the validation and test sets and were excluded from the training set. Therefore, both the validation and test sets covered seven categories, namely paraquat, thiram, tricyclazole, paraquat–thiram binary mixtures, thiram–tricyclazole binary mixtures, paraquat–tricyclazole binary mixtures, and paraquat–thiram–tricyclazole ternary mixtures, with 50 samples per category in the validation set and 53 samples per category in the test set.

### 3.4. Overall Framework

This study addresses SERS mixture recognition under pure-substance spectral training. Let $x\in R^{L}$ denote a preprocessed SERS spectrum, and let the candidate pure components be paraquat, thiram, and tricyclazole, denoted as A, B, and C, respectively. Accordingly, the pure-substance categories are A, B, and C, whereas the binary and ternary mixture categories are AB, AC, BC, and ABC.

For a pure spectrum, the class label is denoted by y∈{1,…,K}. For a mixed spectrum, its composition is represented by a binary indicator vector $z\in \{0,1{\}}^{K}$, where $z_{k}=1$ indicates the presence of the k-th component. The corresponding mixture category is expressed as a set S⊆{1,…,K}. In this work, model optimization is performed using only pure spectra, whereas mixture spectra are introduced only at the validation and test stages for checkpoint selection and final evaluation. The objective is to determine, for an input spectrum x, the most probable mixture category $\hat{S}$.

The framework comprises a spectral encoder, a pure-spectrum classification branch, a component evidence branch, and a two-stage mixture inference module. Given an input spectrum x, the encoder extracts a latent representation

(1) $$f=F\left(x\right),\;f\in R^{d},$$

where F(⋅) denotes the feature extraction network and d is the embedding dimension. The latent feature is then passed to two parallel branches. The first preserves discriminative structure among the pure components, whereas the second outputs component-wise evidence that is transformed into probabilities for downstream inference.

Final recognition is performed in two stages. First, the predicted component probabilities are used to evaluate predefined binary candidate sets and select the most probable binary category. Second, the input spectrum is compared with binary and ternary reconstructions generated from pure-component prototypes, and a residual-based rule determines whether the initial binary prediction should be retained or upgraded to a ternary category. In this way, the framework links pure-spectrum learning to mixture-category recognition without mixture-specific parameter training.

### 3.5. Spectral Encoder and Dual-Branch Learning

#### 3.5.1. ResNet1D Spectral Encoder

The shared spectral encoder is implemented as a one-dimensional residual convolutional network consisting of an initial 1D convolutional block, several residual blocks, and a global average pooling layer. The convolutional layers capture local spectral responses, whereas the residual architecture improves gradient propagation and stabilizes higher-level feature extraction [26]. The pooled output serves as the shared latent representation for both downstream branches.

#### 3.5.2. Pure-Spectrum Classification Branch

The pure-spectrum classification branch is designed to preserve discriminative structure among the K pure components in the latent space. To enhance inter-class separability, a large-margin cosine formulation is adopted [27]. By imposing an explicit margin on the target class, this branch promotes class-separable latent features and provides stable supervision for the shared encoder.

#### 3.5.3. Component Evidence Branch

The component evidence branch transforms the shared latent representation into component-wise evidence for mixture recognition [28]. It outputs a K-dimensional evidence vector, which is converted into component probabilities through a sigmoid transformation,

(2) $$p_{k}=\sigma \left(u_{k}\right)=\frac{1}{1+exp\left(-u_{k}\right)},\;k=1,\ldots ,K.$$

where $u_{k}$ denotes the evidence score of the k-th component and $p_{k}$ is the corresponding probability. Under pure-spectrum supervision, the target component is encouraged to yield high evidence, whereas non-target components are suppressed. Together, the two branches enable the encoder to support both pure-spectrum discrimination and component-aware mixture inference.

### 3.6. Two-Stage Inference for Mixture Recognition

Based on the component probability vector predicted by the evidence branch, the final mixture category is determined through a two-stage inference procedure. Rather than introducing an additional classifier over mixture labels, the proposed method first evaluates binary candidate categories using component-level probabilities and then refines the prediction through residual-based ternary verification.

In the first stage, inference is performed over the predefined binary candidate categories. For each candidate set S, the predicted component probabilities are combined into a set-likelihood score that favors high probabilities for included components and low probabilities for excluded components. For numerical stability, the score is computed in the log domain as

(3) $$Score(S)=\sum_{k\in S}\;log\;p_{k}+\sum_{k\notin S}\;log\left(1-p_{k}\right).$$

where $p_{k}$ denotes the predicted probability of the k-th component. The binary candidate with the highest score is selected as the initial prediction.

To determine whether a ternary explanation is more appropriate, the input spectrum is further reconstructed under the selected binary hypothesis and the ternary hypothesis including all candidate components. Reconstruction is performed using pure-component prototype spectra derived from the training set, with nonnegative least-squares fitting to ensure physically meaningful nonnegative contributions. The relative fitting improvement is defined as

(4) $$g=\frac{e_{bin}-e_{tri}}{e_{bin}},$$

where $e_{bin}$ and $e_{tri}$ denote the reconstruction errors under the binary and ternary hypotheses, respectively. To avoid reassignment based only on a marginal numerical gain, the fitting weight of the component absent from the initial binary prediction is further normalized by the total ternary fitting weight to obtain a missing-component ratio, denoted by ρ.

**Figure 8:**
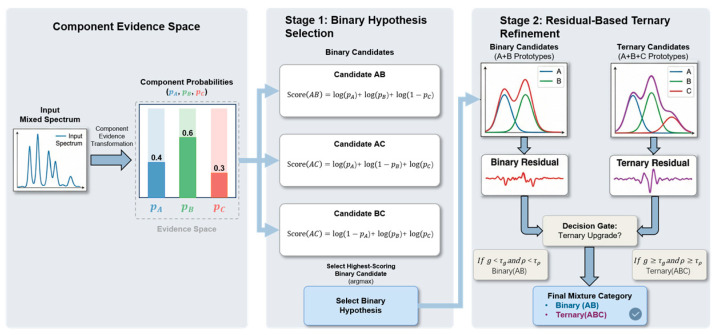
Illustration of the two-stage inference procedure for mixture recognition.

The final prediction is determined jointly by g and ρ:

(5) $$\hat{S}=\left\{\begin{array}{ll} S_{tri}, & g\geq \tau_{g}\;and\;\rho \geq \tau_{\rho },\\ {\hat{S}}_{bin}, & otherwise, \end{array}\right.$$

where ${\hat{S}}_{bin}$ denotes the initial binary prediction, $S_{tri}$ denotes the ternary category, and $\tau_{g}$ and $\tau_{\rho }$ are the corresponding thresholds. As illustrated in Figure 8, this two-stage design combines probabilistic evidence reasoning with residual-based spectral verification, thereby enabling binary and ternary mixture recognition without mixture samples for parameter training.

### 3.7. Training Strategy

**Figure 9:**
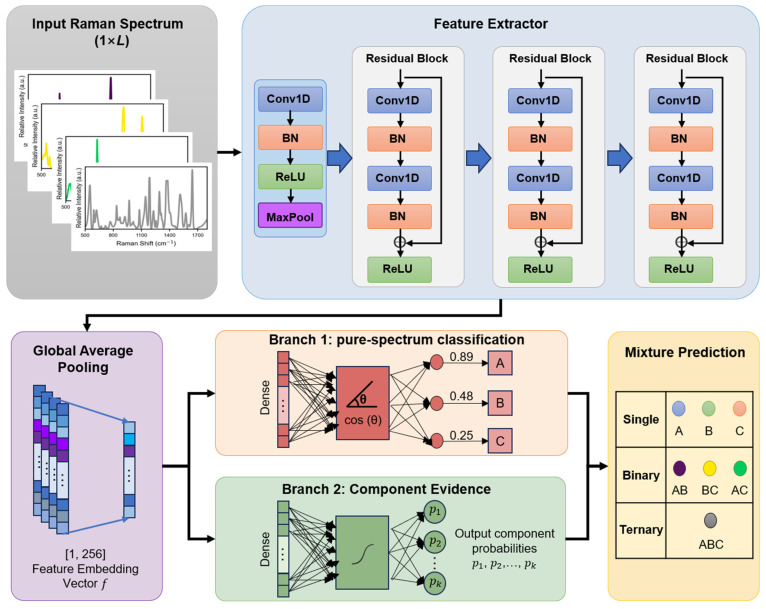
The network architecture of our proposed framework.

Based on the proposed network architecture shown in Figure 9, model optimization was performed using only pure SERS spectra. Paraquat, thiram, and tricyclazole were denoted as A, B, and C, respectively, and the corresponding mixture categories were denoted as AB, AC, BC, and ABC. The training set contained only pure-component spectra, whereas mixture spectra were excluded from parameter optimization and introduced only in the validation and test stages. Under this setting, the model learned transferable spectral representations and component-level evidence from pure-spectrum supervision alone.

The training objective consisted of a pure-spectrum classification loss, a component evidence loss, and an evidence regularization term. The pure-spectrum classification branch was optimized using cross-entropy loss, whereas the component evidence branch was optimized using binary cross-entropy loss with one-hot component indicators. To stabilize the evidence branch and prevent excessively large evidence responses, a clipping-based regularization term was imposed on the raw evidence vector. The total loss is written as

(6) $$L=L_{cls}+\lambda_{evi}L_{evi}+\lambda_{reg}L_{reg},$$

where $L_{cls}$, $L_{evi}$, and $L_{reg}$ denote the classification, evidence, and regularization terms, respectively, and $\lambda_{evi}$ and $\lambda_{reg}$ are the corresponding weighting coefficients. In the present study, $\lambda_{evi}=1.0$ and $\lambda_{reg}=0.01$.

All network parameters were jointly optimized end-to-end using AdamW. The initial learning rate was set to $1\times 10^{-3}$, the weight decay to $1\times 10^{-4}$, and the batch size to 32. A cosine annealing learning-rate schedule was adopted, with the minimum learning rate fixed at $1\times 10^{-5}$. Unless otherwise specified, the embedding dimension was 128, the cosine margin in the pure-spectrum classification branch was 0.35, the classification scaling factor was 16.0, the evidence scaling factor was 16.0, and the evidence clipping threshold was 6.0. The total number of training steps was 3000.

For the residual-based ternary refinement used during inference, pure-component prototype spectra were constructed as class-wise mean spectra from the pure-spectrum training subset. In the present implementation, the relative residual-gain threshold and the missing-component ratio threshold were set to 0.040 and 0.080, respectively.

Model selection followed a validation-based strategy. The pure-substance validation subset was used to monitor pure-spectrum classification performance, whereas the held-out seven-category validation set was used for checkpoint selection. This validation set covered A, B, C, AB, AC, BC, and ABC, but was not used for parameter optimization. To avoid unstable early-stage selection, checkpoint tracking was activated only after 500 training steps, and only checkpoints with a pure-spectrum validation accuracy of at least 0.95 were considered eligible. Among these, the checkpoint achieving the highest recognition accuracy on the seven-category validation set was retained as the final model. This protocol was adopted because optimal pure-spectrum classification does not necessarily imply optimal downstream mixture-recognition performance.

All experiments were implemented in Python 3.10 using PyTorch 2.2 and NumPy 2.2.5. Model training and inference were conducted on an NVIDIA GeForce RTX 3090 GPU with CUDA 12.1.

## 4. Conclusions

In this study, a pure-spectrum-trained framework for SERS mixture recognition was developed and evaluated using paraquat, thiram, and tricyclazole. By combining pure-spectrum representation learning, component evidence modeling, and two-stage inference, the method enabled direct recognition of binary and ternary mixtures without using mixture samples for parameter training. Under the present experimental conditions, the model achieved perfect classification on the pure-spectrum test set and an overall mixture recognition accuracy of 98.58% on the binary and ternary mixture test set. These results indicate that pure-spectrum-trained representations can support mixture recognition in this specific three-component system, and the proposed framework may be regarded as a proof-of-concept method for SERS mixture recognition from pure-substance training.

## Figures and Tables

**Table 1:** Performance metrics for pure-spectrum classification.

Task	Class	Accuracy	Precision	Recall	F1-Score
Pure Substance	Overall	1.0	1.0	1.0	1.0
Pure Substance	Paraquat	-	1.0	1.0	1.0
Pure Substance	Thiram	-	1.0	1.0	1.0
Pure Substance	Tricyclazole	-	1.0	1.0	1.0

**Table 2:** Performance metrics for mixture recognition.

Task	Class	Accuracy	Precision	Recall	F1-Score
Mixtures	Overall	0.9858	0.9863	0.9858	0.9858
Mixtures	AB (Paraquat + Thiram)	-	0.9815	1.0	0.9907
Mixtures	AC (Paraquat + Tricyclazole)	-	1.0	0.9434	0.9709
Mixtures	BC (Thiram + Tricyclazole)	-	1.0	1.0	1.0
Mixtures	ABC (Paraquat + Thiram + Tricyclazole)	-	0.9636	1.0	0.9815

## Data Availability

The raw data supporting the conclusions of this article will be made available by the authors on request.

## Supplementary Materials

The following supporting information can be downloaded at https://www.mdpi.com/article/10.3390/molecules31091412/s1. Figure S1: SERS test data of tricyclazole. Figure S2: SERS test data of thiram. Figure S3: SERS test data of paraquat. Figure S4: SERS test data of tricyclazole and thiram. Figure S5: SERS test data of tricyclazole and paraquat. Figure S6: SERS test data of thiram and paraquat. Figure S7: SERS test data of thiram, tricyclazole and paraquat. Figure S8: SERS test data of thiram, tricyclazole and paraquat. Table S1: The characteristic vibration mode attribution of paraquat. Table S2: The characteristic vibration mode attribution of thiram. Table S3: The characteristic vibration mode attribution of tricyclazole.
