# Supplementary material for: SERS Mixture Recognition from Pure-Substance Spectra via Component Evidence Learning and Two-Stage Inference
Source: Molecules. 2026 Apr 24;31(9):1412. doi: 10.3390/molecules31091412 (PMC13164983; doi:10.3390/molecules31091412)
Supplement: Supplementary file 1 [file molecules-31-01412-s001.zip › molecules-4261115-supplementary.pdf]

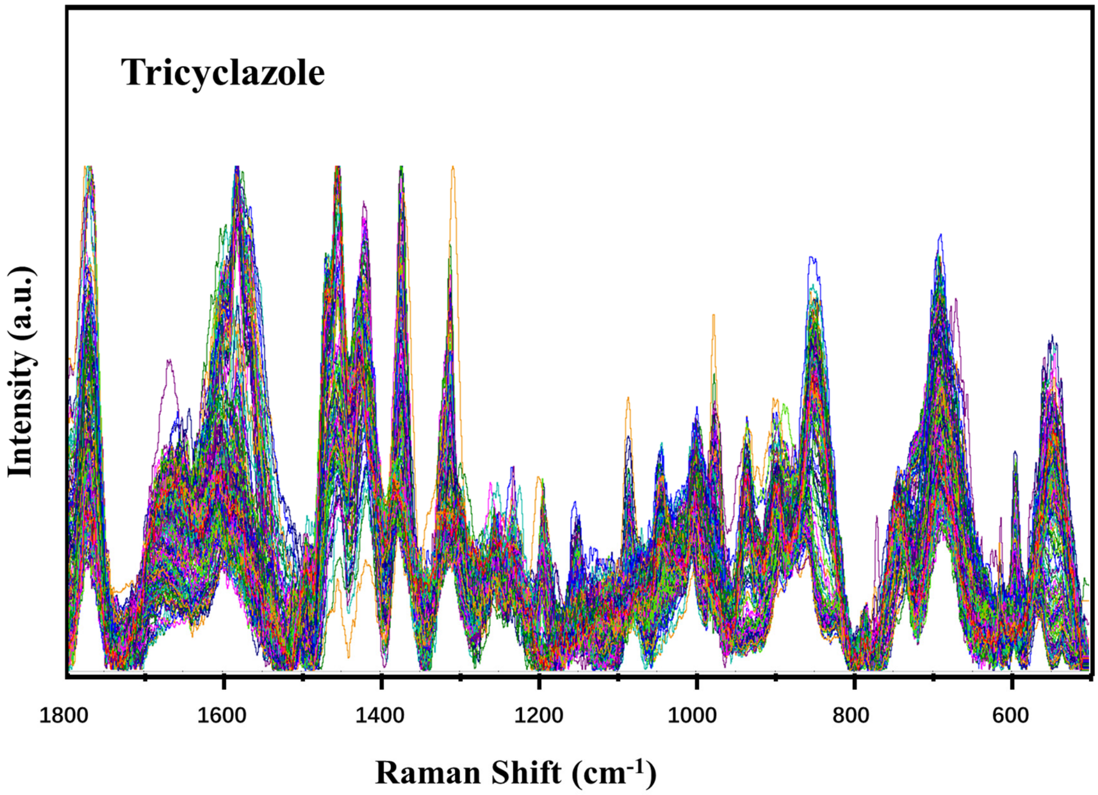

Figure S1 SERS test data of tricyclazole.

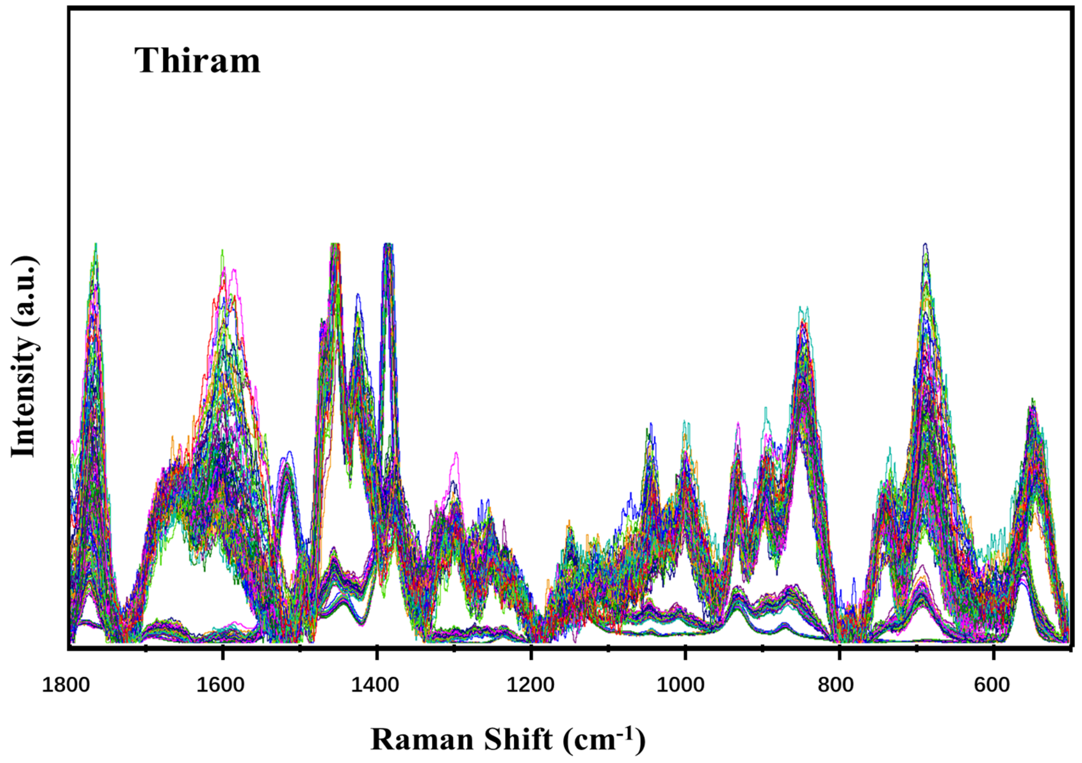

Figure S2 SERS test data of thiram.

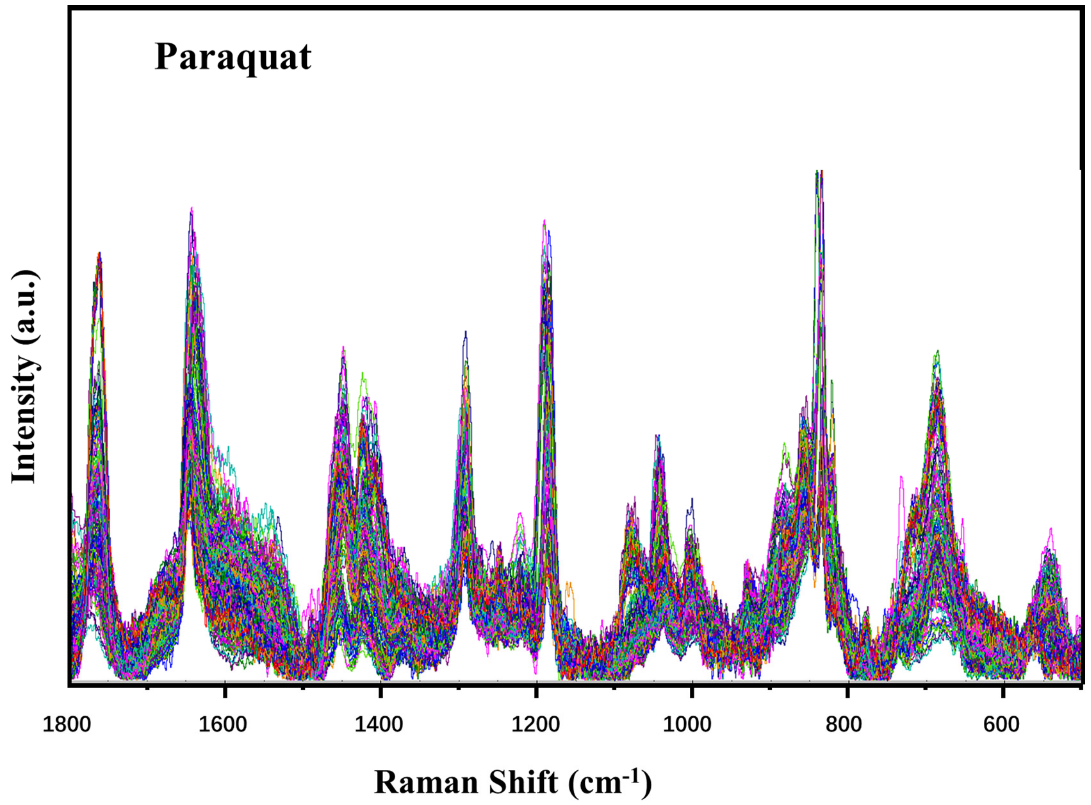

Figure S3 SERS test data of paraquat.

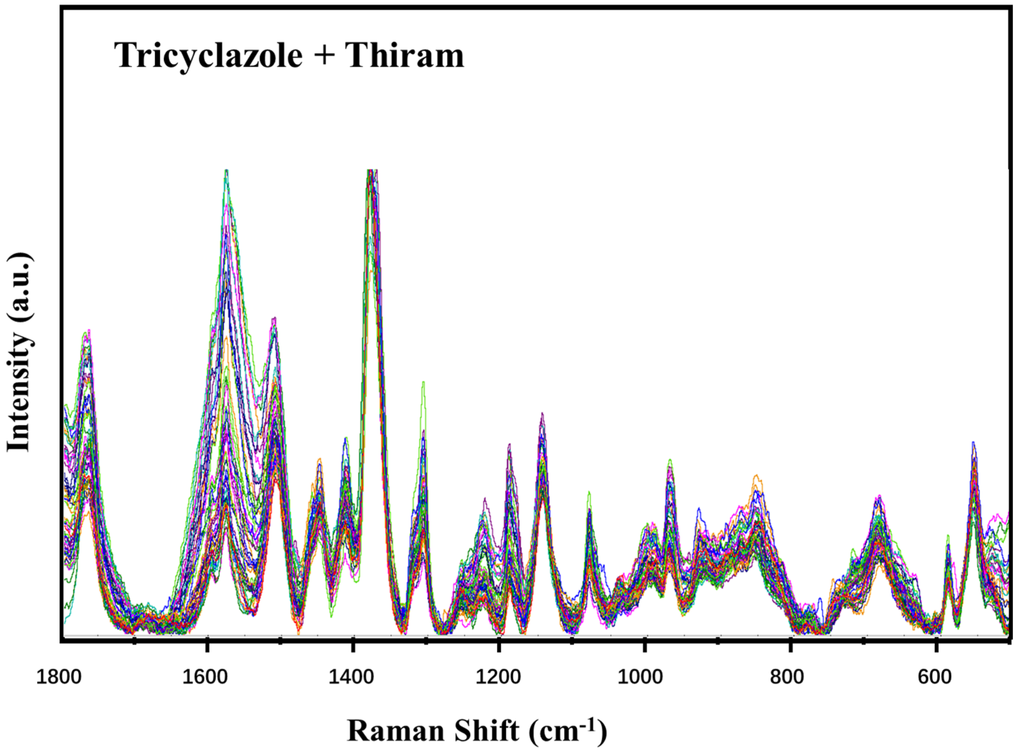

Figure S4 SERS test data of tricyclazole and thiram.

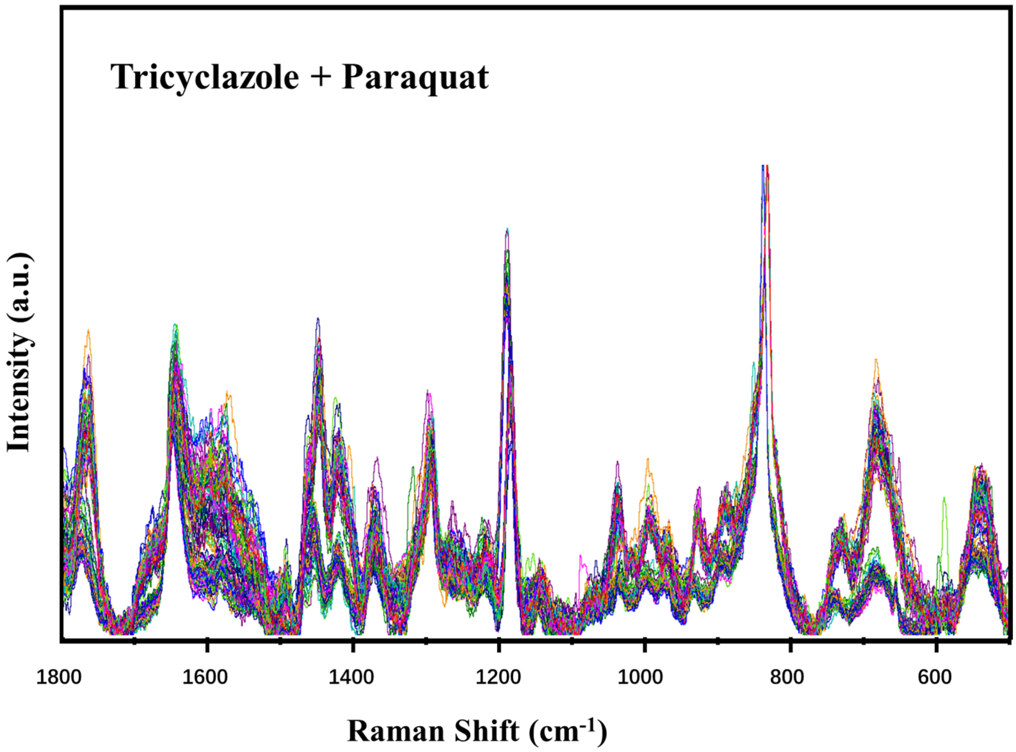

Figure S5 SERS test data of tricyclazole and paraquat.

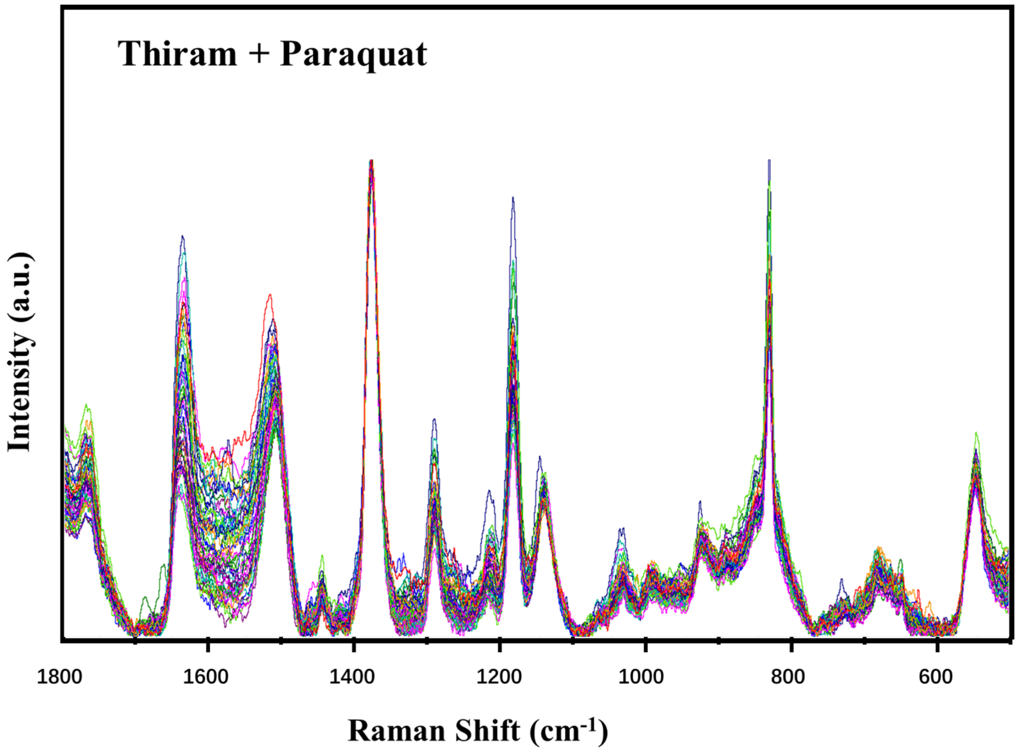

Figure S6 SERS test data of thiram and paraquat.

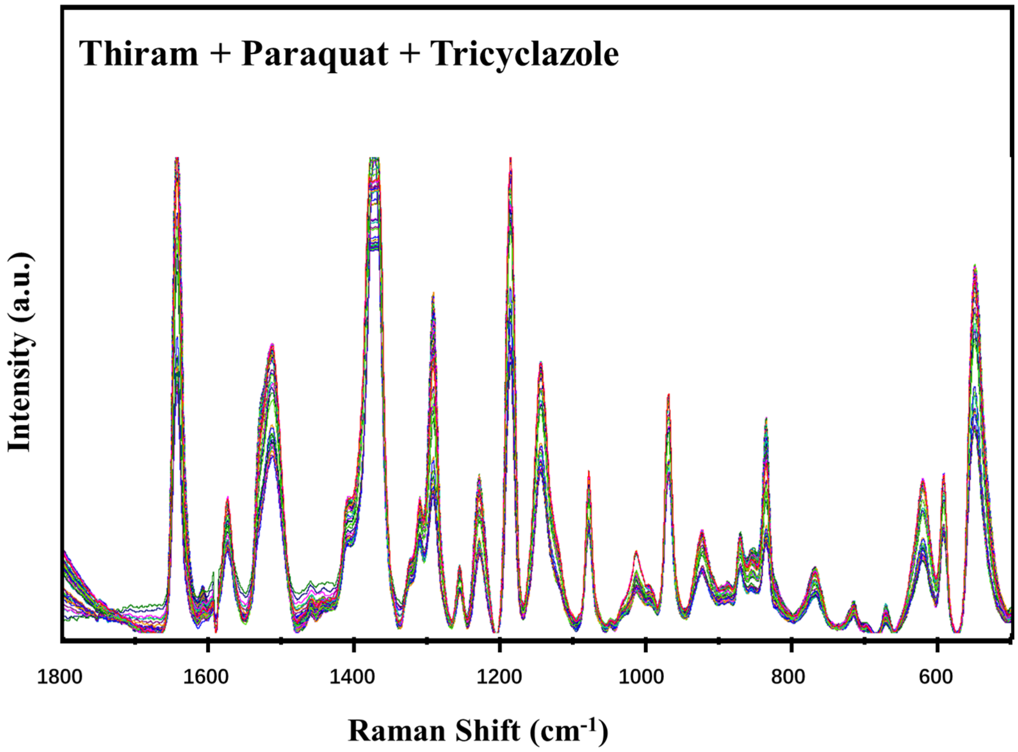

Figure S7 SERS test data of thiram, tricyclazole and paraquat.

Table S1 The characteristic vibration mode attribution of paraquat.

Paraquat	SERS	DFT x 0.986
β(ring) $_{pyridinium}$,ν(C-N)	840	836
ρ(C=C) $_{pyridinium}$	1029	1029
ρ(C–H) $_{pyridinium}$	1190	1185
β(C–H) $_{pyridinium}$	1298	1303
β(C–H) $_{pyridinium}$,ν(C=C)	1646	1635
ν(C=C) $_{pyridinium}$	1766	1766

Table S2 The characteristic vibration mode attribution of thiram.

Thiram	SERS	DFT x 0.967
ρ(C=S) , ρ(C-S) , ρ(C=N)	560	568
ν(C-S) , ν(C-N)	862	860
ν(C=S) , ν(C-N)	932	945
ρ(C-H)	1150	1150
ν(C=N) , ρ(C-H)	1380	1381
ρ(C-H)	1512	1511

Table S3 The characteristic vibration mode attribution of tricyclazole.

Tricyclazole	SERS	DFT x 0.979
β (ring) $_{benzene}$	596	581
ρ(C=N) , ρ(N-N)	941	941
β (ring) $_{benzene}$, β(ring)$_{triazole}$	988	984
ν(N-N) , ρ(C-H)	1000	998
β (ring) $_{benzene}$, ρ(C-H)	1090	1090
ρ(C-H) $_{benzene}$, ν(C-C)	1193	1193
ρ(C-H) $_{benzene}$, ν(C=N)	1303	1300
β(ring)$_{triazole}$, ρ(C-H) $_{benzene}$	1373	1373
ρ(C-H)	1420	1420
ν(C=C)$_{benzene}$, β (C-H)	1589	1600

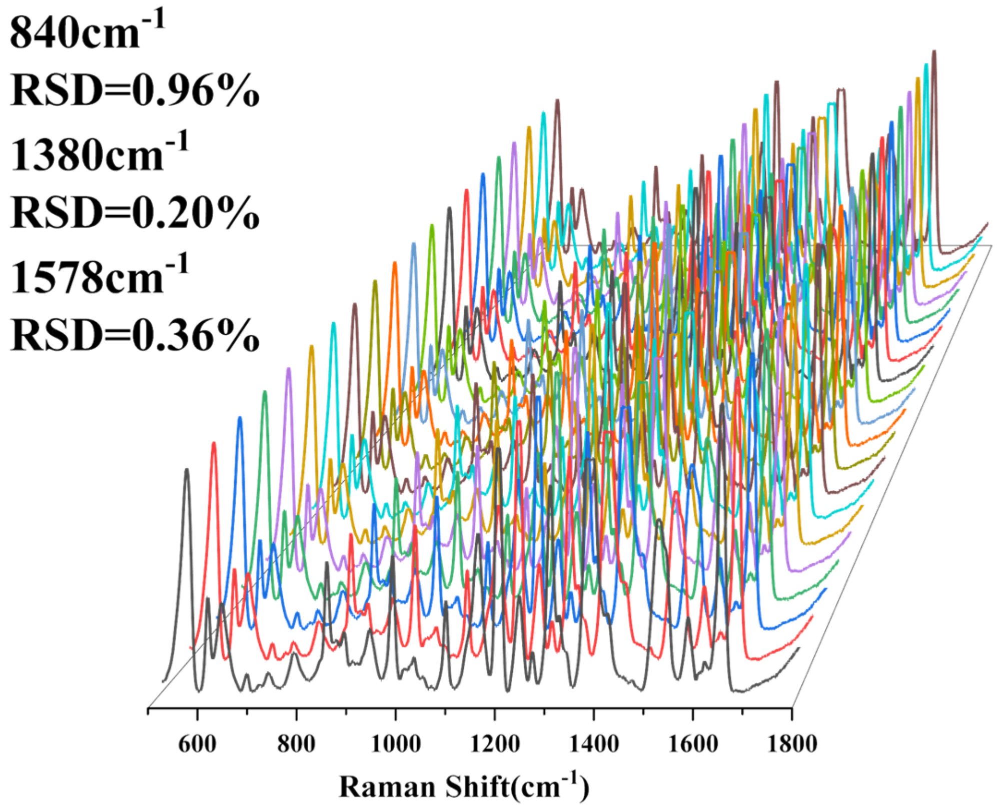

Figure S8 SERS test data of thiram, tricyclazole and paraquat.
